# Comparative study between poorly differentiated thyroid cancer and anaplastic thyroid cancer: real-world pathological distribution, death attribution, and prognostic factor estimation

**DOI:** 10.3389/fendo.2024.1347362

**Published:** 2024-03-13

**Authors:** Kun Zhang, Xinyi Wang, Tao Wei, Zhihui Li, Jingqiang Zhu, Ya-Wen Chen

**Affiliations:** ^1^ Division of Thyroid Surgery, Department of General Surgery, West China Hospital, Sichuan University, Chengdu, Sichuan, China; ^2^ Department of Otolaryngology, Icahn School of Medicine at Mount Sinai, New York, NY, United States; ^3^ Department of Cell, Developmental and Regenerative Biology, Icahn School of Medicine at Mount Sinai, New York, NY, United States; ^4^ Black Family Stem Cell Institute, Icahn School of Medicine at Mount Sinai, New York, NY, United States; ^5^ Institute for Airway Sciences, Icahn School of Medicine at Mount Sinai, New York, NY, United States; ^6^ Center for Epithelial and Airway Biology and Regeneration, Icahn School of Medicine at Mount Sinai, New York, NY, United States

**Keywords:** poorly differentiated thyroid carcinoma, anaplastic thyroid carcinoma, pathological distribution, prognostic factor, death attribution

## Abstract

**Background:**

The clinic-pathological boundary between poorly differentiated thyroid cancer (PDTC) and anaplastic thyroid cancer (ATC) is unclear due to a wide spectrum of histopathological features and the rarity of the disease. In addition to that, with the highest mortality rate and non-standard treatment modality, the PDTC/ATC population has not been subjected to comprehensive description and comparison with the extent of histological characteristics, therapeutic response, prognostic factors, and death attribution analysis.

**Method:**

A total of 4,947 PDTC/ATC patients from 2000 to 2018 were identified from the Surveillance, Epidemiology, and End Results (SEER) database. Kaplan–Meier survival curve estimation and Cox proportional hazard regression were applied.

**Results:**

Overall, the 5- and 10-year DSS for PDTC were 71.9% and 68.0%, respectively, whereas the 5- and 10-year OS are 59.3% and 51.2%, respectively. The median survival time for ATC patients was 3 months with 1-year OS being 26.9% and 1-year DSS being 31.2%. During the follow-up period, 68.1% of the PDTC/ATC cohort were dead, 51.6% of which were attributed to thyroid malignancies and 16.5% to non-thyroid causes. The top three common non-thyroid causes of death were miscellaneous cancers, lower respiratory system disease, and heart disease. The histological feature of papillary thyroid cancer (PTC) was the leading pathological category for PDTC patients (51.7%), whereas 76.7% of ATC patients’ pathological feature was characterized as unidentifiable. Sarcoma histological characteristics found in ATC cases suffer the highest overall mortality (vs. PTC, HR = 2.61, 95% CI 1.68–4.06, P < 0.001). Older age unidentifiable histology feature, more advanced AJCC N1b, AJCC M1, and SEER stage, tumor size larger than 5 cm, and more invasive tumor extension were independent bad outcome predictors.

**Conclusion:**

The populational analysis of the PDTC/ATC cohort has provided reliable support for better understanding of the difference between PDTC and ATC cases and the guidance of clinical practice and further studies.

## Introduction

Conceptually speaking, PDTC and ATC are both considered to be carcinomas originating from the follicular thyroid epithelium that retain an insufficient degree of differentiation in the morphologic characteristics of oncocytes and other histological features (1–7). However, there was a long-standing dispute regarding the appropriate pathological definition of PDTC, ever since Sakamoto et al. and Carcangiu et al. initially described its pathological characteristics in 1983 (1) and 1984 (2). In 2006, the Turin proposal (3) summarized pathological diagnostic criteria, which included (1) the presence of a solid/trabecular/insular pattern of growth (2); the absence of the conventional nuclear features of papillary carcinoma; and (3) the presence of at least one of the following features: convoluted nuclei, mitotic activity in greater than or equal to 3 out of 10 high-power fields, and tumor necrosis. Despite these efforts, PDTC still remains a clinically and pathologically inchoate disorder, and it is still not known whether it represents a single clinical or molecular entity.

The histopathological spectrum of ATC is also highly variable (4, 5), which creates diagnostic challenges and reflects the disease’s underlying genetic complexity. Despite this diversity, a number of subtypes, such as sarcomatoid, giant, and squamoid, have been recognized (6, 7). Adding more complexity to the pathology of ATC, morphological alterations such as mixed chronic inflammation are usually present in ATC (8, 9). The differential diagnosis of ATC includes a large group of non-follicular cell-derived tumors (4). For instance, true sarcomas of the thyroid can be notoriously difficult to distinguish from sarcomatoid types of ATC (10). In some cases, patients with resected differentiated carcinoma (DTC) or PDTC are more likely to develop ATC, sometimes in tandem with DTC. Collectively, the histopathological diversities of ATCs are presumed to be the results of preexisting DTCs or PDTCs (11–14). Meanwhile, squamous cell carcinoma of the thyroid, anaplastic forms of medullary thyroid cancer (MTC), primary thyroid lymphoma (PTL), and a board spectrum of miscellaneous tumors are entities in need of differential diagnosis from ATC (15).

Despite the complexity of their histological characteristics, there is significant disparity in survival outcomes between PDTC and ATC populations, underscoring the critical importance of accurate pathological diagnose. With a reported incidence from 2% to 15% of all thyroid cancers and a 5-year DSS of 66%, PDTC is less aggressive and more common (16, 17) than ATC, which accounts for 1%–2% of all thyroid cancers (18) with 1-year survival approximately 20% (19, 20) and a 3–6-month median survival time.

In brief, histological confirmation of PDTC and ATC is highly complex and largely dependent on rarity of the disease, changing criteria of diagnostics, pathologist’s experience, and subjectivity (16). As a result, true boundaries between these two entities remain vague. Furthermore, histopathological subtypes of PDTC and ATC might exist, each with a different survival outcome. Given its high mortality rate, the population of PDTC and ATC might be the optimal subject regarding the study of death attribution in all thyroid-originated malignancies. A large cohort of PDTC and ATC patients has not been subjected to comprehensive description and comparison to date, due to the rarity of these diseases. Thus, we sought to acquire a cohort of PDTC and ATC patients from the SEER Program to present demographics, real-world pathological distributions, death attributions, and prognostic factor estimation, with the goal of generating a systemic demonstration and comparison between PDTC and ATC patients.

## Materials and methods

### Data sources

Our study utilized a cohort of pathologically confirmed PDTC and ATC patients from the SEER program, a public database that provides information on patient demographics, tumor morphology, stage at diagnosis, primary tumor site, first course of treatment, and follow-up for vital status and causes of death, to reduce the cancer burden among the U.S. population. SEER included 18 population-based cancer registries across the U.S. that comprise approximately 27.8% of the nation’s population (21). No institutional review board approval was required since SEER is an open-access public database with a deidentified dataset. The current study was reported in agreement with the statement of “STROBE” guidelines (22). Our selected database, in accordance with our previous studies (23, 24), is cited as: “Incidence - SEER Research Plus Data, 18 Registries, Nov 2020 Sub (2000–2018) - Linked To County Attributes - Total U.S., 1969-2019 Counties, National Cancer Institute, DCCPS, Surveillance Research Program, released April 2021, based on the November 2020 submission.”

### Study population

Using the selected database, we identified and defined a consecutive cohort of 4,947 patients of primary thyroid malignancy with pathological grading being “poorly differentiated” defined as the PDTC population and “undifferentiated” defined as the ATC population between 2000 and 2018, together as a cohort for analysis. The variable “pathology” referred to in the context of this study is defined as the pathological diagnose made by the SEER database. The clinical data were extracted regarding demographics, tumor staging, and therapeutic approaches: age, gender, race, primary site of tumor, pathology, SEER stage [defined by the SEER database referred to as the SEER stage in our article that represents the staging schema based on information about primary site, histology, or other factors (25)], primary surgery, neck dissection, radiotherapy, chemotherapy, systemic therapy, survival months, cause of death, and survival status.

### Descriptive statistics

Visualizations of pathological distribution and specific cause-of-death illustrations were presented with treemapping (26), doughnut chart, and circular bar plot. The R package involved in the figure layout includes “treemapify”, “tidyverse”, and “ggplot2”.

### Outcome definition

Cause of death was specifically recorded whether each patient died as a result of PDTC/ATC or any other causes (e.g., other cancers, heart disease, pneumonia), or whether they were alive at the end of the follow-up period. In our study, the primary outcome was OS and DSS, which was defined as the time between initial diagnosis and all-cause deaths (for OS) or malignancy-specific deaths (for DSS).

### Cox proportional hazard survival analysis

Univariate Cox proportional hazards regression and Kaplan–Meier curves were used to determine prognostic factors. Significant factors screened by the univariate analysis (P < 0.05) were subsequently included in multivariate Cox proportional hazards models. Therefore, race was not included either in DSS or in OS Cox regression. The best Cox regression model was selected by a backward selection process (entry criterion: P < 0.05, elimination criterion: P > 0.10). Multivariate Cox proportional hazards regression analysis was performed to identify variables that significantly affected the DSS and OS of patients with PDTC/ATC.

### Statistical analysis

We presented descriptive statistics in **Table 1** for the entire study cohort and compared the results between PDTC and ATC patients. Continuous and categorical variables were assessed with the Kruskal–Wallis test and Pearson chi-square test, respectively. Continuous variables were expressed as the mean ± standard deviation (SD)/median. Categorical variables were shown as number (percentage). All statistical analyses were carried out employing the R studio version 4.0.4. A two-tailed P < 0.05 was considered statistically significant.

**Table 1:** Demographic and clinical characteristics of PDTC and ATC patients.

	Overall	PDTC	ATC	P-value
N	4947	2448	2499	
**Age, mean ± SD/median**	64.2 ± 15.9/67	59.3 ± 17.2/61	69.0 ± 12.8/71	<0.001
**Sex, N (%)**				0.575
Female	2,978 (60.2%)	1,464 (59.8%)	1,514 (60.6%)	
Male	1,969 (39.8%)	984 (40.2%)	985 (39.4%)	
**Race, N (%)**				0.025
White	3,933 (79.5%)	1,916 (78.3%)	2,017 (80.7%)	
Black	432 (8.7%)	240 (9.8%)	192 (7.7%)	
^a^Others	582 (11.8%)	292 (11.9%)	290 (11.6%)	
**Pathology, N (%)**				<0.001
PTC	1,733 (35.0%)	1,270 (51.9%)	463 (18.5%)	
FTC	333 (6.7%)	284 (11.6%)	49 (2.0%)	
HCC	120 (2.4%)	89 (3.6%)	31 (1.2%)	
MTC	119 (2.4%)	94 (3.8%)	25 (1.0%)	
Sarcoma	23 (0.5%)	6 (0.2%)	17 (0.7%)	
PTL	6 (0.1%)	5 (0.2%)	1 (0.04%)	
Germ cell and trophoblastic	5 (0.1%)	5 (0.2%)	0 (0.0%)	
Unidentified	2,608 (52.7%)	695 (28.4%)	1,913 (76.6%)	
**SEER stage, N (%)**				<0.001
Localized	917 (18.5%)	744 (30.4%)	173 (6.9%)	
Regional	1,185 (24.0%)	678 (27.7%)	507 (20.3%)	
Distant	1,834 (37.1%)	546 (22.3%)	1,288 (51.5%)	
Unstaged	1,011 (20.4%)	480 (19.6%)	531 (21.2%)	
**Tumor size, N (%)**				<0.001
≤1 cm	140 (2.8%)	112 (4.6%)	28 (1.1%)	
>1 cm and ≤2cm	298 (6.0%)	223 (9.1%)	75 (3.0%)	
>2 cm and ≤3cm	347 (7.0%)	250 (10.2%)	97 (3.9%)	
>3 cm and <=4cm	357 (7.2%)	201 (8.2%)	156 (6.2%)	
>4 cm and ≤5cm	387 (7.8%)	199 (8.1%)	188 (7.5%)	
>5 cm	1,331 (26.9%)	528 (21.6%)	803 (32.1%)	
Unspecified	2087 (42.2%)	935 (38.2%)	1,152 (46.1%)	
**Tumor extension, N (%)**				<0.001
Confined to thyroid capsule	1,059 (21.4%)	778 (31.8%)	281 (11.2%)	
Strap muscle	565 (11.4%)	388 (15.8%)	177 (7.1%)	
Thyroid cartilage or Esophagus	337 (6.8%)	107 (4.4%)	230 (9.2%)	
Trachea or bone	507 (10.2%)	162 (6.6%)	345 (13.8%)	
RLN or vagus nerve	73 (1.5%)	28 (1.1%)	45 (1.8%)	
Major blood vessel	206 (4.2%)	64 (2.6%)	142 (5.7%)	
Mediastinal or Prevertebral fascia	416 (8.4%)	83 (3.4%)	333 (13.3%)	
Unspecified	1,784 (36.1%)	838 (34.2%)	946 (37.9%)	
**Surgery, N (%)**				<0.001
No surgery	1,443 (29.2%)	318 (13.0%)	1,125 (45.0%)	
Lobectomy	350 (7.1%)	170 (6.9%)	180 (7.2%)	
Total thyroidectomy	2,753 (55.6%)	1,801 (73.6%)	952 (38.1%)	
Other surgery	401 (8.1%)	159 (6.5%)	242 (9.7%)	
**Neck dissection, N (%)**				<0.001
No	2,402 (48.6%)	1,040 (42.5%)	1,362 (54.5%)	
Yes	1,840 (37.2%)	1,077 (44.0%)	763 (30.5%)	
Unspecified	705 (14.3%)	331 (13.5%)	374 (15.0%)	
**Radiotherapy, N (%)**				<0.001
No/unknown	1,996 (40.3%)	916 (37.4%)	1,080 (43.2%)	
RAI	1,207 (24.4%)	1,068 (43.6%)	139 (5.6%)	
EBRT	1,744 (35.3%)	464 (19.0%)	1,280 (51.2%)	
**Chemotherapy, N (%)**				<0.001
No/unknown	3,811 (77.0%)	2,224 (90.8%)	1,587 (63.5%)	
Yes	1,136 (23.0%)	224 (9.2%)	912 (36.5%)	
**Systemic therapy, N (%)**				<0.001
No	1,950 (39.4%)	825 (33.7%)	1,125 (45.0%)	
After surgery	1,445 (29.2%)	863 (35.3%)	582 (23.3%)	
Before and after surgery	52 (1.1%)	35 (1.4%)	17 (0.7%)	
Unspecified	1,500 (30.3%)	725 (29.6%)	775 (31.0%)	
**Cause of deaths, N (%)**				<0.001
Alive	1,577 (31.9%)	1,302 (53.2%)	275 (11.0%)	
Thyroid	2,554 (51.6%)	695 (28.4%)	1,859 (74.4%)	
Other causes	816 (16.5%)	451 (18.4%)	365 (14.6%)	
**Survival months, mean ± SD/median**	41.5 ± 56.0/12.0	65.3 ± 60.5/46.0	18.2 ± 39.2/3.0	<0.001

a Others, American Indian/Alaska Native, Asian/Pacific Islander; PTC, papillary thyroid cancer; FTC, follicular thyroid cancer; HCC, Hürthle cell cancer; MTC, medullary thyroid cancer; PTL, primary thyroid lymphoma; SEER, the Surveillance, Epidemiology, and End Results program; SEER stage: see materials and methods; RLN, recurrent laryngeal nerve; RAI, radioactive iodine; EBRT, external beam radiation therapy; SD, standard deviation.

## Results

**Figure 1:**
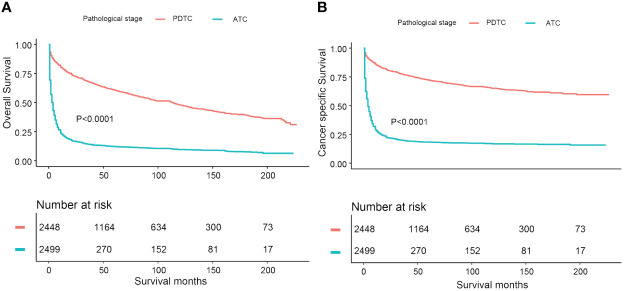
Comparative Kaplan-Meier curves illustrating overall survival and disease-specific survival of PDTC and ATC patients: **(A)** Kaplan-Meier curve estimating overall survival; **(B)** Kaplan-Meier curve estimating disease-specific survival.

In this study, a cohort of 4,958 consecutive primary thyroid malignant tumor patients with “poorly differentiated” or “undifferentiated” pathological grades was identified using the selected SEER database from 2000 to 2018. There were 11 patients excluded from the cohort due to a lack of survival time records. In total, 4,947 PDTC/ATC patients were incorporated in our analysis. The population with PDTC/ATC exhibited these characteristics: diagnosed at middle–old age (median age 67 years old, range from 5 to 85); mostly white (3,933, 79.5%); and slightly female predominant (the male-to-female sex ratio is approximately 2 to 3). The median follow-up time of the study cohort was 12 months (range from 1 month to 227 months). The total number of deaths during the follow-up period was 3,370 (68.1%), of which 2,554 (51.6%) were attributed to thyroid malignancy and 816 (16.5%) to non-thyroid causes, rendering a cancer-specific death dominant attribution. Overall, the 5- and 10-year DSS for PDTC were 71.9% (CI: 70.1%–73.6%) and 68.0% (CI: 66.1%–70.1%), respectively, whereas the 5- and 10-year OS were 59.3% (CI: 57.5%–61.2%) and 51.2% (CI: 49.1%–53.4%), respectively. The median survival time for ATC patients was 3 months, with 1-year OS being 26.9% (CI: 25.3%–28.6%) and 1-year DSS being 31.2% (CI: 29.3%–33.1%). Kaplan–Meier curves estimating OS and DSS for PDTC and ATC patients are illustrated in **Figure 1**.

**Figure 2:**
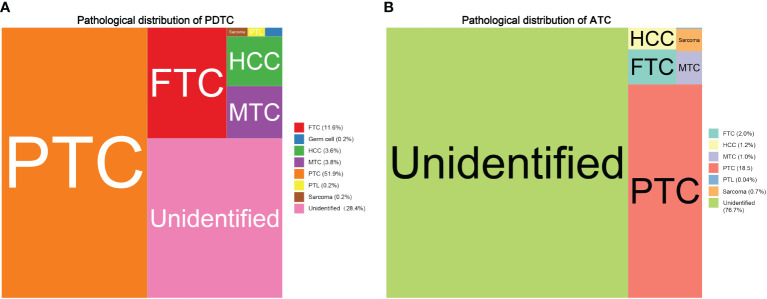
Treemaps comparing the main pathohistological features distribution between PDTC and ATC patients: **(A)** pathological distribution of PDTC; **(B)** pathological distribution of ATC.

To statistically describe and compare the cohort of PDTC and ATC patients, demographics, tumor characteristics, therapeutic approaches, follow-up, and outcome associated variables were all taken into consideration. Compared with PDTC patients, the ATC population was a group of people approximately 10 years older (PDTC vs. ATC: 59.3 ± 17.2 vs. 69.0 ± 12.8, P < 0.001), had no significant difference in sex ratio (P = 0.575), and was borderline different in ethnicity proportion (ATC had a slightly higher white to black ratio, P = 0.025). The most dramatic contrast between the two groups was that the median survival time of the PDTC population was 46 months, whereas it was merely 3 months in ATC patients. In terms of tumor characteristics, based on the methodology of the SEER combined staging system (25), approximately 30% of PDTC patients (744, 30.4%) were classified as local stage, more than 25% (678, 27.7%) as regional stage, and approximately 20% (546, 22.3%) as distant stage. Meanwhile, the SEER stage proportion in the ATC cohort was as follows: nearly 7% as local stage (173, 6.9%); roughly 20% as regional stage (507, 20.3%); and more than 50% as distant stage (1288, 51.5%). Confirmed pathological diagnoses were vastly different in proportion between PDTC and ATC populations. As shown in **Figure 2**, PTC was the leading diagnosis for PDTC patients (51.7%), whereas 76.7% of ATC patients had unidentifiable pathological features. Tumor size at diagnosis was also statistically distinguishable between PDTC and ATC patients. The ATC population’s tumor size was generally larger than 5 cm (≥5 cm, ATC vs. PDTC: 32.1% vs. 21.6%) compared with PDTCs. Tumor extension of the PDTC patients tended to be less aggressive than that of the ATC subcohort, as 47.5% of the tumors were either confined to thyroid capsule (778, 31.8%) or invasive to strap muscle (388, 15.8%). In contrast, more advanced local regional involvements (thyroid cartilage or esophagus, trachea or bone, recurrent laryngeal or vagus nerve, and major blood vessels) were at least twice as frequent in ATC patients, compared with PDTC patients. Invasion of the mediastinum or prevertebral fascia was three times as common in the ATC population as in the PDTC population (ATC vs. PDTC: 13.3% vs. 3.4%). In terms of treatments, the vast majority of PDTC patients were given total thyroidectomy (1801, 73.6%), whereas only 38.1% of the ATC subcohort had total thyroidectomy. Nearly half (1125, 45.0%) of ATC cases were inoperable. Traditional chemotherapy (ATC vs. PDTC: 36.5% vs. 9.2%) and external beam radiation (ATC vs. PDTC: 51.2% vs. 19.0%) therapy were more commonly used for ATC patients, in contrast to PDTC patients. Radioactive iodine therapy had been broadly used in 43.6% of PDTC patients. The percentage in ATC populations was a mere 5.6%.

**Figure 3:**
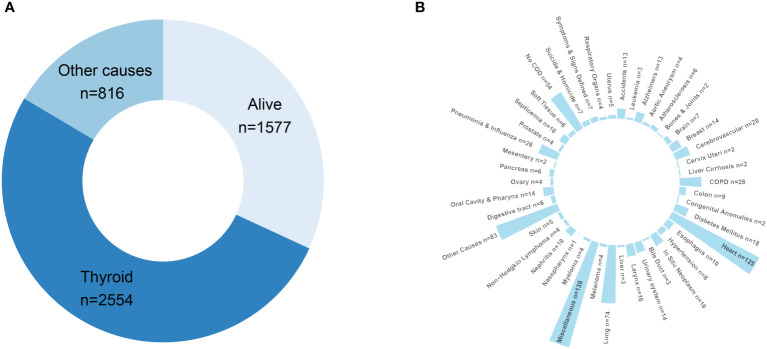
Death attribution of the PDTC/ATC population: **(A)** doughnut chart demonstrating the final outcome of the study cohort at the end of the follow-up; **(B)** circular bar plot specifying the causes-of-death in “other causes”.

Cause of death distribution is illustrated in **Figure 3**. At the end of the follow-up period, 52% of PDTC/ATC patients had died of thyroid malignancy, and 16.5% had died from other causes. To specifically describe the causes of death in “other causes,” we have plotted a circular bar plot in **Figure 3B** showing that there are 57 categories of death causes available in the SEER program. According to the data, the top 10 most common causes of death were miscellaneous malignant tumor (138 cases), diseases of heart (125 cases), lung and bronchus disease (74 cases), chronic obstructive pulmonary disease (28 cases), pneumonia and influenza (26 cases), cerebrovascular disease (20 cases), diabetes mellitus (18 cases), larynx disease (16 cases), breast disease (14 cases), and tied for the 10th place Alzheimer’s disease (13 cases), accidents and adverse effects (13 cases), and kidney and renal pelvis disease (13 cases).

In the multivariate Cox analysis estimating DSS, older age (each increase in 1 year, HR = 1.02, 95% CI 1.02–1.03, P < 0.001), unidentifiable histology features (vs. PTC, HR = 1.54, 95% CI 1.38–2.83, P < 0.001), worse pathological grade (ATC vs. PDTC, HR = 2.54, 95% CI 2.29–2.83, P < 0.001), and higher SEER stage (e.g., distant vs. localized, HR = 5.40 95% CI 4.29–6.80, P < 0.001). More invasive tumor extension (e.g., thyroid cartilage or esophagus vs. confined to thyroid capsule, HR = 1.49, 95% CI 1.22–1.82, P < 0.001) were clinically significant worse survival prognostic factors, after adjustment and model selection from the univariate Cox regression. Surgery (e.g., total thyroidectomy vs. no surgery, HR = 0.47, 95% CI 0.42–0.53, P < 0.001), external beam radiation therapy (EBRT vs. no/unknown, HR = 0.74, 95% CI 0.67–0.81, P < 0.001), chemotherapy (vs. no/unknown, HR = 0.82, 95% CI 0.74–0.91, P < 0.001), and systemic therapy (e.g., post-surgery therapy vs. no, HR = 0.86, 95% CI 0.76–0.98, P < 0.001) were statistically capable of predicting better DSS outcome statistically. Race, gender, and neck dissection were not significantly relevant to DSS in the multivariate regression model. However, in the univariate analysis for DSS, chemotherapy (vs. no/unknown, HR = 2.00, 95% CI 1.84–2.18, P < 0.001) and EBRT (vs. no/unknown, HR = 1.26, 95% CI 1.16–1.37, P < 0.001) were estimated to be detrimental predictors, contrary to that in the multivariate Cox model. Neck dissection (vs. not performed, HR = 0.57, 95% CI 0.52–0.62, P < 0.001) was found to be a significant therapeutic predictor in the univariate regression. **Table 2** provides detailed statistics about DSS.

**Table 2:** Univariate and multivariate Cox proportional hazard regression for analyses of PDTC and ATC patients for disease-specific survival.

	Multivariate			Univariate		
	HR	95% CI	P value	HR	95% CI	P value
**Age (year)**	1.02	(1.02–1.03)	<0.001	1.04	(1.04–1.05)	<0.001
Sex						
Female	1	Reference		1	Reference	
Male				1.06	(0.98–1.15)	0.118
Race						
White	1	Reference		1	Reference	
Black				0.91	(0.79–1.05)	0.199
^a^Others				0.97	(0.86–1.10)	0.683
Pathology						
PTC	1	Reference		1	Reference	
FTC	1.13	(0.91–1.39)	0.267	1.08	(0.96–1.33)	0.474
HCC	0.86	(0.63–1.18)	0.358	1.08	(0.96–1.55)	0.610
MTC	0.90	(0.67–1.21)	0.486	1.39	(1.06–1.72)	0.024
Sarcoma	1.15	(0.59–2.23)	0.688	2.85	(3.50–8.34)	0.002
PTL	0.00	(0.00–inf.)	0.971	NA	(0.00–inf.)	0.971
Germ cell and trophoblastic	1.24	(0.31–5.03)	0.761	1.07	(0.27–4.31)	0.920
Unidentified	1.54	(1.38–1.73)	<0.001	4.20	(3.81–4.63)	<0.001
Pathological grade						
PDTC	1	Reference		1	Reference	
ATC	2.54	(2.29–2.83)	<0.001	5.46	(4.99–5.97)	<0.001
SEER stage						
Localized	1	Reference		1	Reference	
Regional	2.53	(2.00–3.21)	<0.001	4.04	(3.30–4.94)	<0.001
Distant	5.40	(4.29–6.80)	<0.001	13.11	(10.83–15.86)	<0.001
Unstaged	2.21	(1.66–2.94)	<0.001	5.98	(4.89–7.31)	<0.001
Tumor size						
≤1cm	1	Reference		1	Reference	
>1 cm and ≤2 cm	1.22	(0.75–1.99)	0.427	1.29	(0.79–2.10)	0.304
>2 cm and ≤3 cm	1.31	(0.82–2.08)	0.259	1.97	(1.24–3.13)	0.004
>3 cm and ≤4 cm	1.44	(0.92–2.27)	0.112	3.14	(2.01–4.92)	<0.001
>4 cm and ≤5 cm	1.60	(1.02–2.50)	0.040	4.03	(2.59–6.26)	<0.001
>5 cm	1.98	(1.29–3.05)	0.002	6.62	(4.34–10.12)	<0.001
Unspecified	2.11	(1.36–3.28)	0.001	5.59	(3.66–8.52)	<0.001
Tumor extension						
Confined to thyroid capsule	1	Reference		1	Reference	
Strap muscle	1.15	(0.93–1.41)	0.192	1.81	(1.50–2.17)	<0.001
Thyroid cartilage or Esophagus	1.49	(1.22–1.82)	<0.001	4.78	(3.99–5.74)	<0.001
Trachea or bone	1.12	(0.92–1.36)	0.262	5.84	(4.97–6.87)	<0.001
RLN or vagus nerve	1.40	(1.01–1.93)	0.043	3.87	(2.85–5.27)	<0.001
Major blood vessel	1.38	(1.10–1.73)	0.005	5.78	(4.71–7.08)	<0.001
Mediastinal or prevertebral fascia	1.02	(0.84–1.24)	0.834	8.44	(7.13–9.99)	<0.001
Unspecified	0.99	(0.80–1.21)	0.904	3.50	(3.04–4.03)	<0.001
Surgery						
No surgery	1	Reference		1	Reference	
Lobectomy	0.58	(0.49–0.69)	<0.001	0.29	(0.24–0.34)	<0.001
Total thyroidectomy	0.47	(0.42–0.53)	<0.001	0.16	(0.15–0.17)	<0.001
Other surgery	0.65	(0.56–0.76)	<0.001	0.39	(0.34–0.45)	<0.001
Neck dissection						
No	1	Reference		1	Reference	
Yes	1.04	(0.93–1.16)	0.525	0.57	(0.52–0.62)	<0.001
Unspecified	1.51	(1.27–1.79)	<0.001	0.89	(0.80–100)	0.045
Radiotherapy						
No/unknown	1	Reference		1	Reference	
RAI	0.46	(0.39–0.54)	<0.001	0.20	(0.18–0.23)	<0.001
EBRT	0.74	(0.67–0.81)	<0.001	1.26	(1.16–1.37)	<0.001
Chemotherapy						
No/unknown	1	Reference		1	Reference	
Yes	0.82	(0.74–0.91)	<0.001	2.00	(1.84–2.18)	<0.001
Systemic therapy						
No	1	Reference		1	Reference	
After surgery	0.86	(0.75–0.98)	0.019	0.44	(0.40–0.49)	<0.001
Before and after surgery	1.15	(0.73–1.80)	0.557	0.44	(0.28–0.68)	<0.001
Unspecified	1.01	(0.89–1.14)	0.866	0.71	(0.64–0.77)	<0.001

HR, hazard ratio; CI, confidential interval. ^a^Others, American Indian/Alaska Native, Asian/Pacific Islander; PTC, papillary thyroid cancer; FTC, follicular thyroid cancer; HCC, Hürthle cell cancer; MTC, medullary thyroid cancer; PTL, primary thyroid lymphoma; SEER, the Surveillance, Epidemiology, and End Results program; SEER stage: see Materials and Methods; RLN, recurrent laryngeal nerve; RAI, radioactive iodine; EBRT, external beam radiation therapy; SD, standard deviation.

Most independent prognostic factors found in the univariate and multivariate Cox regression for OS predicted the same trends as the DSS regression with different hazard ratios (detailed in **Table 3**). There were some new findings and exceptions in the OS Cox regression. For instance, male gender predicted an added 13% probability of death in multivariate Cox models. Thyroid cartilage/esophagus invasion (OS, vs. within capsule, HR = 1.28, P = 0.005; DSS, vs. within capsule, HR = 1.49, P < 0.001) and major blood vessel involvement (OS, vs. within capsule, HR = 1.28, P = 0.013; DSS, vs. within capsule, HR = 1.38, P < 0.005) can also effectively predict decreased OS and DSS in univariate and multivariate Cox models. Histological features of sarcoma (vs. PTC, HR = 2.61, 95% CI 1.68–4.06, P < 0.001) was associated with an increased risk of overall death in the multivariate Cox analysis. Neck dissection (vs. not performed, P = 0.479) and systemic therapy (variable not included by backward selection process) were not statistically significant in predicting OS in the univariate and multivariate Cox regression models separately.

**Table 3:** Univariate and multivariate Cox proportional hazard regression for analyses of PDTC and ATC patients for overall survival.

	Multivariate			Univariate		
	HR	95% CI	P value	HR	95% CI	P value
**Age (year)**	1.03	(1.03–1.03)	<0.001	1.05	(1.04–1.05)	<0.001
Sex						
Female	1	Reference		1	Reference	
Male	1.13	(1.05–1.21)	0.001	1.12	(1.04–1.20)	0.001
**Race**						
White	1	Reference		1	Reference	
Black				1.00	(0.88–1.13)	0.972
^a^Others				0.92	(0.83–1.03)	0.173
Pathology						
PTC	1	Reference		1	Reference	
FTC	1.10	(0.93–1.30)	0.275	1.13	(0.96–1.33)	0.139
HCC	0.95	(0.74–1.21)	0.650	1.22	(0.96–1.55)	0.105
MTC	0.84	(0.66–1.08)	0.175	1.35	(1.06–1.72)	0.015
Sarcoma	2.61	(1.68–4.06)	<0.001	5.41	(3.50–8.34)	<0.001
PTL	0.25	(0.06–1.01)	0.051	0.53	(0.13–2.14)	0.375
Germ cell and trophoblastic	0.85	(0.21–3.44)	0.824	0.73	(0.18–2.92)	0.654
Unidentified	1.52	(1.38–1.67)	<0.001	3.78	(3.48–4.10)	<0.001
Pathological grade						
PDTC	1	Reference		1	Reference	
ATC	2.10	(1.92–2.29)	<0.001	4.23	(3.93–4.55)	<0.001
SEER stage						
Localized	1	Reference		1	Reference	
Regional	1.90	(1.59–2.27)	<0.001	2.68	(2.32–3.10)	<0.001
Distant	3.67	(3.09–4.37)	<0.001	7.84	(6.85–8.96)	<0.001
Unstaged	1.63	(1.31–2.02)	<0.001	3.59	(3.11–4.15)	<0.001
Tumor size						
≤1 cm	1	Reference		1	Reference	
>1 cm and ≤2 cm	1.07	(0.74–1.54)	0.718	1.09	(0.76–1.56)	0.650
>2 cm and ≤3 cm	1.12	(0.79–1.58)	0.517	1.62	(1.15–2.28)	0.005
>3 cm and ≤4 cm	1.22	(0.87–1.71)	0.240	2.35	(1.68–3.27)	<0.001
>4 cm and ≤5 cm	1.31	(0.94–1.82)	0.115	2.91	(2.10–4.03)	<0.001
>5 cm	1.59	(1.15–2.18)	0.004	4.57	(2.91–5.38)	<0.001
Unspecified	1.85	(1.33–2.56)	<0.001	3.96	(2.91–5.38)	<0.001
Tumor extension						
Confined to thyroid capsule	1	Reference		1	Reference	
Strap muscle	1.12	(0.94–1.33)	0.197	1.56	(1.35–1.81)	<0.001
Thyroid cartilage or Esophagus	1.28	(1.08–1.52)	0.005	3.50	(3.00–4.07)	<0.001
Trachea or bone	1.05	(0.89–1.24)	0.544	4.25	(3.72–4.86)	<0.001
RLN or vagus nerve	1.30	(0.98–1.74)	0.071	2.86	(2.18–3.75)	<0.001
Major blood vessel	1.28	(1.05–1.56)	0.013	4.32	(3.63–5.15)	<0.001
Mediastinal or prevertebral fascia	0.99	(0.84–1.18)	0.930	6.46	(5.62–7.45)	<0.001
Unspecified	0.88	(0.74–1.05)	0.145	2.67	(2.39–2.98)	<0.001
Surgery						
No surgery	1	Reference		1	Reference	
Lobectomy	0.50	(0.43–0.58)	<0.001	0.27	(0.24–0.31)	<0.001
Total thyroidectomy	0.43	(0.39–0.47)	<0.001	0.16	(0.15–0.17)	<0.001
Other surgery	0.61	(0.53–0.69)	<0.001	0.37	(0.33–0.42)	<0.001
Neck dissection						
No	1	Reference		1	Reference	
Yes	0.97	(0.88–1.06)	0.479	0.56	(0.52–0.61)	0.356
Unspecified	1.39	(1.20–1.61)	<0.001	0.84	(0.76–0.93)	<0.001
Radiotherapy						
No/unknown	1	Reference		1	Reference	
RAI	0.47	(0.42–0.53)	<0.001	0.24	(0.22–0.27)	<0.001
EBRT	0.71	(0.66–0.77)	<0.001	1.17	(1.09–1.26)	<0.001
Chemotherapy						
No/unknown	1	Reference		1	Reference	
Yes	0.77	(0.70–0.84)	<0.001	1.80	(1.68–1.96)	<0.001
Systemic therapy						
No	1	Reference		1	Reference	
After surgery				0.45	(0.41–0.49)	<0.001
Before and after surgery				0.48	(0.33–0.69)	<0.001
Unspecified				0.69	(0.63–0.74)	<0.001

HR, hazard ratio; CI, confidential interval. ^a^Others, American Indian/Alaska Native, Asian/Pacific Islander; PTC, papillary thyroid cancer; FTC, follicular thyroid cancer; HCC, Hürthle cell cancer; MTC, medullary thyroid cancer; PTL, primary thyroid lymphoma; SEER, the Surveillance, Epidemiology, and End Results program; SEER stage: see Materials and Methods; RLN, recurrent laryngeal nerve; RAI, radioactive iodine; EBRT, external beam radiation therapy; SD, standard deviation.

## Discussion

ATC and PDTC are rare populations, but clinically significant due to the fact that they account for the majority of thyroid malignancy deaths (27). Moreover, the clinicopathologic boundaries separating ATC from PCTC are difficult to define in clinical practice (28). Therefore, an in-depth comparison and characterization is needed. The significance of summarizing the demographic and clinical characteristics of the most lethal population of thyroid cancer is key to the early diagnosis of such patients (28). The PDTC/ATC patients selected in our study generally presented at an older age (median age 67, PDTC median age 61, ATC median age 71) and with a higher male-to-female ratio (3:2, so as in PDTC and ATC separately), which is similar to previous reports (16, 27, 29). Older age and male gender were found to be adverse prognostic factors for OS in our analysis, which is also in accordance with former studies (16, 29, 30), and their presence in PDTC/ATC patients may indicate more aggressive biological behavior. Furthermore, more aggressive than PDTC, ATC presents with a larger size, more invasive extrathyroidal extension, more frequent AJCC M1 stage, and a dominantly higher cancer-specific death rate, as described in previous studies (15, 31).

Since the SEER program designed these two variables, “Grade (thru 2017)” and “AYA site recode 2020 Revision” (32, 33), we were able to define a cohort of PDTC (poorly differentiated, grade III) and ATC (undifferentiated, grade IV) patients and capture pathological diagnostic features shown in **Figure 2**. While we cannot redefine disease entities solely based on basic histological characteristics due to the lack of pathological imaging data, it is critical to highlight the significance of high mitotic activity, tumor necrosis, and specific cytoarchitectural features in distinguishing disease entities. The criteria set by the Memorial Sloan Kettering Cancer Center (MSKCC) (34), which were introduced a year prior to the Turin proposal, include identifying patients who may benefit from aggressive treatments due to a higher risk of distant metastasis, which is predominantly seen in RAS-driven PDTC (35).

The tree map supports the fact that the true histogenesis of PDTC (3, 36) and ATC (7, 37) is highly complex and hard to classify. For instance, the concept of high-grade differentiated thyroid cancer (HG-DTC), characterized by the cytoarchitectural features of well-differentiated thyroid carcinoma, such as papillary structures, but with increased mitotic activity and/or tumor necrosis may fall into the category of the PTC in **Figure 2** (38). In fact, most thyroid sarcoma-like tumors are probably anaplastic carcinomas. Small cell types reported in the past were probably lymphoma or variants of medullary or insular carcinoma or unidentifiable, as shown in our study and previous reports (7, 36). In addition, squamous cell carcinoma of the thyroid is now considered a morphologic pattern/subtype of ATC, according to the 5^th^ edition of the Classification of Endocrine and Neuroendocrine Tumors released by the World Health Organization (38).

To our knowledge, this is the first report of death attribution regarding thyroid cancer. The primary causes of death in the PDTC/ATC population are due to thyroid cancer, as one can expect, followed by miscellaneous tumors, lower respiratory system disease (compounding lung and bronchus, chronic obstructive pulmonary disease, and pneumonia and influenza together), and heart disease (**Figure 3**). The interpretation of the common causes of death can be insightful. For one thing, ventilatory and sputum excretion disorders may result from trachea compression caused by a rapidly growing neck mass, thereby raising pulmonary disease susceptibility. For another, airway obstruction may lead to severe hypoxia and respiratory distress syndrome that eventually result in pulmonary and cardiac failure. In addition, miscellaneous tumors are also a major contributing factor to death, which cannot be ignored. As the SEER program database did not specifically define the terminology “miscellaneous tumor” here, we cited from elsewhere (39) that cancers of unknown primary site, Kaposi sarcoma, neuroblastoma, neuroendocrine tumors, paraganglioma, metastatic cancers, thymoma, etc., may outline this concept for convenience of understanding. In brief, the “miscellaneous” group of PDTC/ATC patents died of a subsequent primary cancer (SPC). There is some evidence that higher-risk SPCs among survivors may be related to genetic factors as well as treatment exposures, but most of the greater excess risk is likely to be caused by host factors (such as aging and compromised immunity) (40, 41). With this in mind, an antiproliferation therapy combining immunotherapy for PDTC/ATC may be encouraged, which is supported by a German clinical study (42).

In terms of regimen modality for the PDTC/ATC population, an unusual divergence in the results of univariate and multivariate Cox regressions for EBRT and chemotherapy has drawn our attention. In the univariate Cox model for OS (EBRT: HR = 1.17, P < 0.001, chemo: HR = 1.80, P < 0.001) and DSS (EBRT: HR = 1.26, P < 0.001, chemo: HR = 2.00, P < 0.001), both EBRT and chemotherapy played an adverse role. After the adjustment and step-wise backward selection process, EBRT and chemotherapy were both estimated to be independent predictors of better OS (EBRT: HR = 0.71, P < 0.001, chemo: HR = 0.76, P < 0.001) and DSS (EBRT: HR = 0.74, P < 0.001, chemo: HR = 0.81, P < 0.001). The interpretation should be made with caution. One should always bear in mind that the study cohort is composed of PDTC and ATC patients, thereby chemo- and radiotherapy were probably administrated to the ATC populations, in keeping with the American Thyroid Association (ATA) guideline (43) and also reflected in our data (see **Table 1**). As a result, most ATC patients have been tagged by chemotherapy and/or radiotherapy and therefore predicted inferior OS and DSS in the univariate Cox regressions. However, the true curative effect of chemo- and radiotherapy, although limited (43), had been revealed after prognostic factor adjustments in the final multivariate Cox models.

Some limitations are undeniable. For instance, the clinical management and their associated therapeutic responses for PDTC and ATC populations may differ substantially, yet we are unable to investigate this based on our cohort. Our research design did not permit a second review of these specimens, introducing a potential bias. This bias arises from the diversity in experience and expertise among the pathologists who was involved in the data source collected by the SEER program. This could impact the consistency and reliability of diagnoses, thereby influencing the study’s outcomes. Acknowledging this limitation is crucial for interpreting our findings. Aside from that, data registry selection bias, presence of confounders, and the retrospective design are inherent limitations to our study.

Except for the above disadvantages, we have characterized the largest cohort to date for the analysis of the most lethal subpopulation of thyroid cancer. The death attribution demonstration, histology-associated survival analysis, and populational evidence-based prognostic factors have provided a better understanding of the difference between PDTC and ATC cases, along with guidance for clinical practice and further studies.

## Data availability statement

We use the SEER database for the study. Researchers can request access from the SEER website to obtain access to the database. Requests to access the datasets should be directed to https://seerdataaccess.cancer.gov/seer-data-access.

## Ethics statement

Ethical approval was not required for the study involving humans in accordance with the local legislation and institutional requirements. Written informed consent to participate in this study was not required from the participants or the participants’ legal guardians/next of kin in accordance with the national legislation and the institutional requirements.

## Author contributions

KZ: Writing – review & editing, Writing – original draft, Supervision, Methodology, Investigation, Formal analysis, Conceptualization. XW: Writing – original draft. TW: Writing – review & editing, Formal analysis. ZL: Writing – review & editing, Supervision, Formal analysis. JZ: Writing – review & editing, Supervision. Y-WC: Writing – review & editing, Supervision, Conceptualization.

## References

[1] Sakamoto A Kasai N Sugano H. Poorly differentiated carcinoma of the thyroid. A clinicopathologic entity for a high-risk group of papillary and follicular carcinomas. Cancer. (1983) 52:1849–55. doi: 10.1002/(ISSN)1097-0142 6313176

[2] Carcangiu ML Zampi G Rosai J. Poorly differentiated ("insular") thyroid carcinoma. A reinterpretation of Langhans' "wuchernde Struma". Am J Surg Pathol. (1984) 8:655–68. doi: 10.1097/00000478-198409000-00005 6476195

[3] Volante M Collini P Nikiforov YE Sakamoto A Kakudo K Katoh R. Poorly differentiated thyroid carcinoma: the Turin proposal for the use of uniform diagnostic criteria and an algorithmic diagnostic approach. Am J Surg Pathol. (2007) 31:1256–64. doi: 10.1097/PAS.0b013e3180309e6a 17667551

[4] Ragazzi M Ciarrocchi A Sancisi V Gandolfi G Bisagni A Piana S. Update on anaplastic thyroid carcinoma: morphological, molecular, and genetic features of the most aggressive thyroid cancer. Int J Endocrinol. (2014) 2014:790834. doi: 10.1155/2014/790834 25214840 PMC4158294

[5] Lampertico P. Anaplastic (sarcomatoid) carcinoma of the thyroid gland. Semin Diagn Pathol. (1993) 10:159–68. 8367624

[6] Talbott I Wakely PE Jr. Undifferentiated (anaplastic) thyroid carcinoma: Practical immunohistochemistry and cytologic look-alikes. Semin Diagn Pathol. (2015) 32:305–10. doi: 10.1053/j.semdp.2014.12.012 25596874

[7] Carcangiu ML Steeper T Zampi G Rosai J. Anaplastic thyroid carcinoma. A study of 70 cases. Am J Clin Pathol. (1985) 83:135–58. doi: 10.1093/ajcp/83.2.135 2578727

[8] Caillou B Talbot M Weyemi U Pioche-Durieu C Al Ghuzlan A Bidart JM. Tumor-associated macrophages (TAMs) form an interconnected cellular supportive network in anaplastic thyroid carcinoma. PloS One. (2011) 6:e22567. doi: 10.1371/journal.pone.0022567 21811634 PMC3141071

[9] Sato T Omura M Saito J Hirasawa A Kakuta Y Wakabayashi Y. Neutrophilia associated with anaplastic carcinoma of the thyroid: production of macrophage colony-stimulating factor (M-CSF) and interleukin-6. Thyroid. (2000) 10:1113–8. doi: 10.1089/thy.2000.10.1113 11201858

[10] Kuhn E Ragazzi M Ciarrocchi A Torricelli F de Biase D Zanetti E. Angiosarcoma and anaplastic carcinoma of the thyroid are two distinct entities: a morphologic, immunohistochemical, and genetic study. Mod Pathol. (2019) 32:787–98. doi: 10.1038/s41379-018-0199-z 30723294

[11] Oishi N Kondo T Ebina A Sato Y Akaishi J Hino R. Molecular alterations of coexisting thyroid papillary carcinoma and anaplastic carcinoma: identification of TERT mutation as an independent risk factor for transformation. Mod Pathol. (2017) 30:1527–37. doi: 10.1038/modpathol.2017.75 28731042

[12] Landa I Ibrahimpasic T Boucai L Sinha R Knauf JA Shah RH. Genomic and transcriptomic hallmarks of poorly differentiated and anaplastic thyroid cancers. J Clin Invest. (2016) 126:1052–66. doi: 10.1172/JCI85271 PMC4767360 26878173

[13] Yoo SK Song YS Park YJ Seo JS. Recent improvements in genomic and transcriptomic understanding of anaplastic and poorly differentiated thyroid cancers. Endocrinol Metab (Seoul). (2020) 35:44–54. doi: 10.3803/EnM.2020.35.1.44 32207263 PMC7090308

[14] Wiseman SM Loree TR Hicks WL Jr. Rigual NR Winston JS Tan D. Anaplastic thyroid cancer evolved from papillary carcinoma: demonstration of anaplastic transformation by means of the inter-simple sequence repeat polymerase chain reaction. Arch Otolaryngol Head Neck Surg. (2003) 129:96–100. doi: 10.1001/archotol.129.1.96 12525202

[15] Bible KC Kebebew E Brierley J Brito JP Cabanillas ME Clark TJ Jr.. American thyroid association guidelines for management of patients with anaplastic thyroid cancer. Thyroid. (2021) 31:337–86. doi: 10.1089/thy.2020.0944 PMC8349723 33728999

[16] Ibrahimpasic T Ghossein R Carlson DL Nixon I Palmer FL Shaha AR. Outcomes in patients with poorly differentiated thyroid carcinoma. J Clin Endocrinol Metab. (2014) 99:1245–52. doi: 10.1210/jc.2013-3842 24512493

[17] Lee DY Won JK Lee SH Park DJ Jung KC Sung MW. Changes of clinicopathologic characteristics and survival outcomes of anaplastic and poorly differentiated thyroid carcinoma. Thyroid. (2016) 26:404–13. doi: 10.1089/thy.2015.0316 26541309

[18] Chintakuntlawar AV Foote RL Kasperbauer JL Bible KC. Diagnosis and management of anaplastic thyroid cancer. Endocrinol Metab Clin North Am. (2019) 48:269–84. doi: 10.1016/j.ecl.2018.10.010 30717908

[19] Smallridge RC Copland JA. Anaplastic thyroid carcinoma: pathogenesis and emerging therapies. Clin Oncol (R Coll Radiol). (2010) 22:486–97. doi: 10.1016/j.clon.2010.03.013 PMC3905320 20418080

[20] Neff RL Farrar WB Kloos RT Burman KD. Anaplastic thyroid cancer. Endocrinol Metab Clin North Am. (2008) 37:525–38 xi. doi: 10.1016/j.ecl.2008.02.003 18502341

[21] Overview of the SEER program-incidence-SEER research plus data, 18 registries (2021). Available online at: https://seer.cancer.gov/data-software/documentation/seerstat/nov2020/.

[22] von Elm E Altman DG Egger M Pocock SJ Gotzsche PC Vandenbroucke JP. The Strengthening the Reporting of Observational Studies in Epidemiology (STROBE) statement: guidelines for reporting observational studies. Lancet. (2007) 370:1453–7. doi: 10.1016/S0140-6736(07)61602-X 18064739

[23] Zhang K Su A Wang X Zhao W He L Wei T. Non-linear correlation between tumor size and survival outcomes for parathyroid carcinoma: A SEER population-based cohort study. Front Endocrinol (Lausanne). (2022) 13:882579. doi: 10.3389/fendo.2022.882579 35846299 PMC9285012

[24] Zhang K Peng X Wei T Li Z Zhu J Chen YW. Prognostic nomogram and competing risk analysis of death for primary thyroid lymphoma: A long-term survival study of 1638 patients. Ann Surg Open. (2022) 3:e226. doi: 10.1097/AS9.0000000000000226 36590887 PMC9780050

[25] SEER combined stage: a cancer staging schema using Collaborative Stage (CS) to collecting stage using the TNM classification . Available online at: https://seer.cancer.gov/seerstat/variables/seer/ajcc-stage/seer-combined.html.

[26] Kong N Heer J Agrawala M. Perceptual guidelines for creating rectangular treemaps. IEEE Trans Vis Comput Graph. (2010) 16:990–8. doi: 10.1109/TVCG.2010.186 20975136

[27] Ibrahimpasic T Ghossein R Shah JP Ganly I. Poorly differentiated carcinoma of the thyroid gland: current status and future prospects. Thyroid. (2019) 29:311–21. doi: 10.1089/thy.2018.0509 PMC6437626 30747050

[28] Xu B Ibrahimpasic T Wang L Sabra MM Migliacci JC Tuttle RM. Clinicopathologic features of fatal non-anaplastic follicular cell-derived thyroid carcinomas. Thyroid. (2016) 26:1588–97. doi: 10.1089/thy.2016.0247 PMC5105347 27480016

[29] Shaha AR Loree TR Shah JP. Intermediate-risk group for differentiated carcinoma of thyroid. Surgery. (1994) 116:1036–40. 7985084

[30] McIver B Hay ID Giuffrida DF Dvorak CE Grant CS Thompson GB. Anaplastic thyroid carcinoma: a 50-year experience at a single institution. Surgery. (2001) 130:1028–34. doi: 10.1067/msy.2001.118266 11742333

[31] Sugitani I Miyauchi A Sugino K Okamoto T Yoshida A Suzuki S. Prognostic factors and treatment outcomes for anaplastic thyroid carcinoma: ATC Research Consortium of Japan cohort study of 677 patients. World J Surg. (2012) 36:1247–54. doi: 10.1007/s00268-012-1437-z 22311136

[32] AYA site recode 2020 revision: the surveillance, epidemiology, and end results program. In: The SEER AYA site recode was developed to better define the major cancer sites that affect individuals between 15 and 39 years of age. It is intended to facilitate SEER's reporting of cancer incidence rates and trends. The variable was added to the SEER databases as a convenience for researchers. Available at: https://seer.cancer.gov/ayarecode/.

[33] Barr RD Holowaty EJ Birch JM. Classification schemes for tumors diagnosed in adolescents and young adults. Cancer. (2006) 106:1425–30. doi: 10.1002/cncr.21773 16544312

[34] Xu B Ghossein R. Poorly differentiated thyroid carcinoma. Semin Diagn Pathol. (2020) 37:243–7. doi: 10.1053/j.semdp.2020.03.003 32360274

[35] Xu B Ghossein RA. Advances in thyroid pathology: high grade follicular cell-derived thyroid carcinoma and anaplastic thyroid carcinoma. Adv Anat Pathol. (2023) 30:3–10. doi: 10.1097/PAP.0000000000000380 36306188

[36] Volante M Landolfi S Chiusa L Palestini N Motta M Codegone A. Poorly differentiated carcinomas of the thyroid with trabecular, insular, and solid patterns: a clinicopathologic study of 183 patients. Cancer. (2004) 100:950–7. doi: 10.1002/cncr.20087 14983490

[37] Sun XS Sun SR Guevara N Fakhry N Marcy PY Lassalle S. Chemoradiation in anaplastic thyroid carcinomas. Crit Rev Oncol Hematol. (2013) 86:290–301. doi: 10.1016/j.critrevonc.2012.10.006 23218594

[38] Christofer Juhlin C Mete O Baloch ZW. The 2022 WHO classification of thyroid tumors: novel concepts in nomenclature and grading. Endocr Relat Cancer. (2023) 30. doi: 10.1530/ERC-22-0293 36445235

[39] Miscellaneous tumors: UpToDate. In: Tumors that do not easily fit into the specific classifications of common tumors. Available at: https://www.uptodate.com/contents/table-of-contents/oncology/miscellaneous-tumors.

[40] Travis LB Demark Wahnefried W Allan JM Wood ME Ng AK. Aetiology, genetics and prevention of secondary neoplasms in adult cancer survivors. Nat Rev Clin Oncol. (2013) 10:289–301. doi: 10.1038/nrclinonc.2013.41 23529000

[41] Sung H Hyun N Leach CR Yabroff KR Jemal A. Association of first primary cancer with risk of subsequent primary cancer among survivors of adult-onset cancers in the United States. JAMA. (2020) 324:2521–35. doi: 10.1001/jama.2020.23130 PMC7756242 33351041

[42] Dierks C Seufert J Aumann K Ruf J Klein C Kiefer S. Combination of lenvatinib and pembrolizumab is an effective treatment option for anaplastic and poorly differentiated thyroid carcinoma. Thyroid. (2021) 31:1076–85. doi: 10.1089/thy.2020.0322 PMC8290324 33509020

[43] Smallridge RC Ain KB Asa SL Bible KC Brierley JD Burman KD. American Thyroid Association guidelines for management of patients with anaplastic thyroid cancer. Thyroid. (2012) 22:1104–39. doi: 10.1089/thy.2012.0302 23130564

